# Could detection and attribution of climate change trends be spurious regression?

**DOI:** 10.1007/s00382-022-06242-z

**Published:** 2022-03-24

**Authors:** Donald P. Cummins, David B. Stephenson, Peter A. Stott

**Affiliations:** 1 Department of Mathematics, University of Exeter, Exeter, UK; 2 Met Office Hadley Centre, Exeter, UK

## Abstract

Since the 1970s, scientists have developed statistical methods intended to formalize detection of changes in global climate and to attribute such changes to relevant causal factors, natural and anthropogenic. Detection and attribution (D&A) of climate change trends is commonly performed using a variant of Hasselmann’s “optimal fingerprinting” method, which involves a linear regression of historical climate observations on corresponding output from numerical climate models. However, it has long been known in the field of time series analysis that regressions of “non-stationary” or “trending” variables are, in general, statistically inconsistent and often spurious. When non-stationarity is caused by “integrated” processes, as is likely the case for climate variables, consistency of least-squares estimators depends on “cointegration” of regressors. This study has shown, using an idealized linear-response-model framework, that if standard assumptions hold then the optimal fingerprinting estimator is consistent, and hence robust against spurious regression. In the case of global mean surface temperature (GMST), parameterizing abstract linear response models in terms of energy balance provides this result with physical interpretability. Hypothesis tests conducted using observations of historical GMST and simulation output from 13 CMIP6 general circulation models produced no evidence that standard assumptions required for consistency were violated. It is therefore concluded that, at least in the case of GMST, detection and attribution of climate change trends is very likely not spurious regression. Furthermore, detection of significant cointegration between observations and model output indicates that the least-squares estimator is “superconsistent”, with better convergence properties than might previously have been assumed. Finally, a new method has been developed for quantifying D&A uncertainty, exploiting the notion of cointegration to eliminate the need for pre-industrial control simulations.

## Introduction

Statistical methods for detection and attribution (D&A) of climate change trends have been widely used in climate change studies over the last two decades, and the resulting inferences have informed assessment reports from the Intergovernmental Panel on Climate Change (IPCC) (Mitchell et al. 2001; Hegerl et al. 2007; Bindoff et al. 2013). Formal D&A studies commonly employ some variant of the method known as “optimal fingerprinting”, introduced by Hasselmann (1979, 1997). Optimal fingerprinting frames D&A as the problem of separating the forced component of historical climate observations (i.e. the signal) from internal climate variability (the noise) (Hegerl and Zwiers 2011). In practice, this separation of signal and noise is performed by projecting climate observations onto corresponding simulation output from general circulation models (GCMs), in a procedure analogous to a multivariate linear regression (Allen and Tett 1999). Optimal fingerprinting assumes a regression model of the form

1 $$\begin{aligned} \varvec{y} = X \varvec{\beta } + \varvec{e}, \end{aligned}$$

where $\varvec{y}$ denotes historical climate observations; *X* is a matrix of predicted climate-change signals, typically consisting of simulation output from a GCM; and $\varvec{e}$ is a composite error term containing internal climate variability noise as well as other sources of uncertainty. Regression coefficients $\varvec{\beta }$ are known in D&A as “scaling factors”. Detection and attribution inferences depend on obtaining reliable estimates of these scaling factors and establishing their statistical significance. Fingerprinting methods have become increasingly sophisticated due to a succession of proposed refinements, e.g. Hegerl et al. (1996), Hegerl et al. (1997), Allen and Tett (1999), Allen and Stott (2003), Huntingford et al. (2006), Ribes et al. (2009), Ribes et al. (2013), Hannart et al. (2014), Hannart (2016), Katzfuss et al. (2017) and Hannart (2019).

While originally conceived as a multivariate method for use with gridded spatio-temporal datasets, typically requiring application of dimension-reduction techniques, simplified variants of optimal fingerprinting have more recently been applied to time series data, specifically to observations of global mean surface temperature (GMST) (Otto et al. 2015; Rypdal 2015; Haustein et al. 2017). Global mean surface temperature is an important climate variable, both as a predictor of changes in local climate (Sutton et al. 2015), and as the metric of global warming used in communication with policymakers, e.g. the 1.5 and 2.0 degrees Celsius global warming limits of the 2015 Paris Agreement. There is also evidence that the additivity assumption (see Sect. 3) implicit in optimal fingerprinting is more likely to hold for GMST than for other variables such as precipitation (Good et al. 2011).

The present study is motivated by an apparent failure in the canonical D&A literature to explicitly recognize the time-indexed nature of climate datasets, an omission which could potentially draw that literature into question. It has long been known in the field of time series analysis that the practice of regressing variables containing time trends comes with a unique set of pitfalls, the most serious of which being the “spurious regression” phenomenon (discussed in detail in Sect. 2). In this paper, the threat to D&A inferences posed by spurious regression will be assessed, using a combination of theoretical argument (Sect. 3) and empirical evidence (Sect. 4). The validity (or otherwise) of optimal fingerprinting studies will be found to depend on a property known as “cointegration”, which may be described informally as a stricter form of correlation arising between time series. Section 4 will also introduce and demonstrate a new method for estimating uncertainty in D&A results, which exploits the notion of cointegration to obviate dependence on pre-industrial control (piControl) simulations (and the strong assumptions therewith).

## Regression of non-stationary variables

When discussing optimal fingerprinting as applied to global temperatures, it is necessary to introduce some definitions from time series analysis, the most important of which being the notion of “stationarity”. In this paper, a time series variable *u*(*t*) is said to be stationary if and only if its mean and variance are finite and do not depend on time *t*. Such a time series exhibits a mean-reverting behaviour. An example of a stationary time series is the first-order autoregressive or AR(1) model

2 $$\begin{aligned} u(t) = \rho u(t-1) + \varepsilon (t), \end{aligned}$$

where $-1< \rho < 1$ is a correlation and $\varepsilon (t)$ a white-noise process, commonly called a “shock” or “innovation”. The AR(1) model in Eq. (2) has mean zero and constant variance $\sigma ^2/(1-\rho ^2)$, where $\sigma ^2=\mathrm {Var}(\varepsilon )$. Stationary autoregressive processes have been proposed as simple models of internal climate variability (Hasselmann 1976). If a trend in a time series can be described as a change in the mean (deterministic trend) or variance (stochastic trend) over time then, by their definition, stationary time series do not exhibit trends.

Detection and attribution is concerned with “trending” (non-stationary) climate variables. While the above definition of stationarity is quite prescriptive, the corresponding class of non-stationary time series, i.e. those violating the conditions, is too broad to be practically useful for the purposes of this paper. Instead, attention will be restricted to the class of non-stationary time series known as “integrated” or “difference-stationary”. For a time series *v*(*t*) to be difference-stationary, the differenced series $\Delta v(t)=v(t)-v(t-1)$ must be stationary. The equivalent term “integrated” comes from the fact that *v*(*t*) can be constructed by integrating (taking partial sums of) the stationary time series $\Delta v(t)$. In this paper, integrated will be abbreviated to I(1), where 1, the “order of integration”, denotes the number of times a series must be differenced to achieve stationarity. An example of an I(1) time series, the simple random walk, can be obtained from Eq. (2) by setting $\rho =1$:

3 $$\begin{aligned} v(t) = v(t-1) + \varepsilon (t). \end{aligned}$$

The variance of *v*(*t*) grows linearly in time, so the series is non-stationary. It may be said that *v*(*t*) contains a “unit root” stochastic trend (see Sect. 3).

The climate variables in D&A studies are hypothesized to be non-stationary, due to the presence of externally forced trends, both natural and anthropogenic. Radiative forcing due to greenhouse gas (GHG) emissions has a natural representation as an integrated process, where the integration is the accumulation of gases in the atmosphere over time. The idea of unit-root stochastic trends has a long history in climate change studies, e.g. Kaufmann and Stern (1997). Stern and Kaufmann (2000), Kaufmann and Stern (2002), Kaufmann et al. (2006), Mills (2008), Kaufmann et al. (2011) and Kaufmann et al. (2013), although it has been disputed (Gay-Garcia et al. 2009). It might further be argued that the processes driving anthropogenic GHG emissions are themselves integrated, where the integration represents accumulation of industrial capacity, however there is no numerical evidence for significant higher-order integration in annual records of historical GMST (see Sect. 4). In optimal fingerprinting, observed and simulated realizations of non-stationary variables are commonly regressed on one another using classical estimators such as ordinary least squares (OLS) (Allen and Tett 1999) or total least squares (TLS) (Allen and Stott 2003), depending on the size of the GCM ensemble. However, it has long been known that regressions involving non-stationary variables are susceptible to a phenomenon called “spurious regression”, whereby statistically significant linear relationships are found between completely unrelated time series (e.g. Yule (1926)). Granger and Newbold (1974) showed that regressing two independent random walks produces inflated *t*-statistics and often leads to the detection of a statistically significant relationship when in reality none exists. In general, OLS regressions of I(1) time series are statistically inconsistent, i.e. the coefficient estimates do not converge in the limit of infinite data, except in the special case where the series are “cointegrated” (Engle and Granger 1987). Two or more I(1) time series are said to cointegrate when there exists a linear combination of the series which is itself stationary. Regressing cointegrated time series using OLS yields coefficient estimates which are not only consistent but “superconsistent”, meaning they converge to the coefficients’ true values at a rate proportional to the length of the series. Thus the question of whether the regressors in optimal fingerprinting are cointegrated is critical for evaluating the reliability of D&A of climate change trends.

The risk of spurious regression in D&A pertains specifically to the attribution problem. This is because, in the case of detection, p-values are calculated “under the null”, i.e. under an assumption of no climate change. In the absence of climate change, the left-hand side of Eq. (1) would be stationary by definition, and there would be no risk of spurious regression. In the case of attribution, where climate change is taken as given, spurious regression refers to the misattribution of climate trends to one or more candidate factors, meaning that the resulting allocation of blame is inaccurate. Such misattribution does not require the presence of an “exogenous” trend (e.g. caused by a hidden forcing mechanism), but may instead be caused by flawed representation of forced trends included in the climate model. In particular, any discrepancy between true and modelled forcing which accumulates over time (e.g. due to inaccurate data/incomplete understanding of physical processes) has the potential to induce non-stationarity in the error term of the regression equation, leading to inconsistent scaling factor estimates and invalid confidence intervals. Given that climate models are known to differ in their representation of radiative forcings, there is a prima facie case for investigating this possibility (Myhre et al. 2013).

Methods based on the notion of cointegration have been used previously in analyses of climatic time series (Bindoff et al. 2013). Much effort has gone into studying cointegrations between groups of real-world variables, such as temperatures and forcings (Stern 2006; Turasie 2012; Beenstock et al. 2012; Stern and Kaufmann 2014; Pretis et al. 2015; Storelvmo et al. 2016; Estrada and Perron 2017; Bruns et al. 2020) or temperatures and sea level (Schmith et al. 2012). However, little effort has gone into discussing the presence or lack of cointegration between observed and model-simulated realizations of the same climate variable.

This study has two primary aims: firstly, to determine mathematically whether the experimental design and model assumptions of optimal fingerprinting together imply cointegration of the regression and therefore consistency of the least squares estimator; secondly, to investigate whether there is empirical evidence of such a cointegration arising in practice for the GMST variable. The first aim will be addressed in Sect. 3 and key results proved within an idealized linear-response-model framework. It will be shown how, by parameterizing the impulse response as an energy-balance model (EBM), the formulas in the proof can assume physical interpretability in terms of real-world quantities. Section 4 deals with the second aim by means of hypothesis testing, applied to historical observations and output from the latest generation of GCMs. A new method for calculating confidence regions without recourse to piControl simulations will also be introduced. The content of the paper is summarized in Sect. 5.

## Theoretical reasons for cointegration

This section will assess the consistency of optimal fingerprinting regression in the presence of I(1) non-stationary forcings. To begin with, some definitions are required.

### Impulse-response model definition

Let *y* denote a climate variable of interest for which historical observations are available. Assuming that a change in *y* in response to an externally imposed effective radiative forcing (ERF) *F* may be adequately described by a linear and time-invariant (LTI) impulse-response function, the time series of observations *y*(*t*) may be written as an autoregressive moving-average (ARMA) model of arbitrary order $p,q \ge 0$,

4 $$\begin{aligned} y(t) - \sum _{i=1}^p \phi _i y(t-i) = c + \sum _{i=0}^q \theta _i F(t-i) + \xi (t), \end{aligned}$$

where $\mu = c/(1-\sum _i \phi _i)$ is variable *y*’s pre-industrial baseline, i.e. its mean value in the absence of any forcing *F*; coefficients $\phi _i$ and $\theta _i$ are sequences of weights determining the autoregressive (AR) and moving-average (MA) parts of the impulse-response function; and $\xi (t)$ is a stationary zero-mean stochastic process representing internal climate variability, plus other sources of noise/uncertainty reasonably considered stationary, such as observational error. The definition of ERF is given in Myhre et al. (2013) and is such that the climate system’s response to ERF should be indifferent to the particular forcing agent responsible. The ARMA model in Eq. (4) is very general, incorporating all finite-impulse-response (FIR) models, as well as all infinite-impulse-response (IIR) models of exponential type. Defining the “backshift operator” $\mathrm {B}$ such that $\mathrm {B}^i x(t) = x(t-i)$, Eq. (4) may be written

5 $$\begin{aligned} y(t) = \mu + \varvec{\Phi }(\mathrm {B}) F(t) + \varepsilon (t), \end{aligned}$$

where the rational function

6 $$\begin{aligned} \varvec{\Phi }(\mathrm {B}) = \dfrac{\varvec{\theta }(\mathrm {B})}{\varvec{\phi }(\mathrm {B})} = \dfrac{\sum _{i=0}^q \theta _i \mathrm {B}^i}{1 - \sum _{i=1}^p \phi _i \mathrm {B}^i}, \end{aligned}$$

is known as the “transfer function”. The time series of radiative forcings *F*(*t*) is assumed to be non-stationary with non-stationarity modelled as I(1),

7 $$\begin{aligned} F(t) = F(t-1) + \Delta F(t), \end{aligned}$$

where the series of forcing increments $\Delta F(t)$ is a stationary stochastic process. The rationale for modelling $\Delta F(t)$ as stochastic is the fact that it cannot be predicted from past values of *F*(*t*) only, as evidenced by the impact on $\text {CO}_2$ emissions of the recent SARS-CoV-2 pandemic (Tollefson 2021). Equation (7) is quite general since, beyond the assumption of stationarity, no specific parametric model is assumed for $\Delta F(t)$. Note that $\Delta F(t)$ need not have zero mean: for example, in the case of forcing due to exponentially increasing atmospheric $\text {CO}_2$ concentration, $\Delta F(t)$ would on average (and indeed almost always) take a positive value. Using the backshift operator,

8 $$\begin{aligned} F(t) = \dfrac{1}{1-\mathrm {B}} \Delta F(t), \end{aligned}$$

whence the term “unit-root” non-stationarity originates, as the polynomial in the transfer function’s denominator contains a unit root. To guarantee the existence of a finite climate sensitivity, it is assumed that all roots of the AR polynomial $\varvec{\phi }(\mathrm {B})$ lie strictly outside the unit circle in the complex plane. Noting that the backshift operator $\mathrm {B}$ reduces to the identity when the system is in equilibrium, the familiar equilibrium climate sensitivity (ECS) for variable *y* is then

9 $$\begin{aligned} y_{\mathrm {ECS}} = \varvec{\Phi }(1) F_{2 \times \text {CO}_2}, \end{aligned}$$

where $F_{2 \times \text {CO}_2}$ denotes the increase in radiative forcing associated with a doubling of atmospheric carbon dioxide $\text {CO}_2$ concentration.

### Optimal fingerprinting experimental design

Consider an optimal fingerprinting study where observed changes in *y* are to be attributed to a set of *p* candidate forcings $F_1, \dots , F_p$. Using the ARMA model in Eq. (4) and the backshift operator notation, the study’s experimental design may be written

10 $$\begin{aligned} y(t)&= \mu + \varvec{\Phi }(\mathrm {B}) F(t) + \varepsilon (t), \end{aligned}$$

11 $$\begin{aligned} x_1(t)&= \mu ' + \varvec{\Phi }'(\mathrm {B}) \pi _1 F_1(t) + \varepsilon '_1(t), \end{aligned}$$

12 $$\begin{aligned}&\vdots \nonumber \\ x_p(t)&= \mu ' + \varvec{\Phi }'(\mathrm {B}) \pi _p F_p(t) + \varepsilon '_p(t). \end{aligned}$$

The equation for observations *y*(*t*) is unchanged. New variables $x_i$ denote output from climate model runs (or ensembles thereof) where forcings $F_i$ have been applied individually. If no important forcing factors are missing from the candidate set, and if there are no interactions between forcing factors, it may be assumed that the total forcing *F* driving the observed trend in *y* has the decomposition $F = F_1 + \dots + F_p$. This is the “additivity assumption” of optimal fingerprinting. Another fundamental assumption of optimal fingerprinting is that radiative forcings driving model runs $x_i$ have the correct temporal structure, i.e. are identical to their real-world counterparts up to multiplicative constants $\pi _i$. In practice this assumption may be relaxed using errors-in-variables (EIV) methods, but at the cost of introducing further assumptions such as model exchangeability (Huntingford et al. 2006). Note that, in Eqs. (11) to (12), the climate model is not assumed to perfectly reproduce the properties of the true climate. In general, the climate model may have a different mean $\mu ' \ne \mu$, a different noise process $\varepsilon ' \ne \varepsilon$, and an impulse response differing from the truth in shape and scale $\varvec{\Phi }' \ne \varvec{\Phi }$.

### Consistency of the least squares estimator

If at least one of the candidate forcings is I(1) non-stationary then a multiple regression of *y* on $x_1, \dots , x_p$ is integrated on both sides of the equation. An integrated regression of this type is known to be consistent if and only if the I(1) variables are cointegrated (Engle and Granger 1987). To establish consistency of optimal fingerprinting, as described by Eqs. (10) to (12), it is therefore necessary and sufficient to prove that *y* and $x_1, \dots , x_p$ cointegrate. From the definition of cointegration, this may be achieved by proving the existence of a linear combination of the $x_i$ which, when subtracted from *y*, yields a stationary process.

#### Lemma

*The rational transfer function* $\varvec{\Phi }(\mathrm {B}) = \varvec{\theta }(\mathrm {B}) / \varvec{\phi }(\mathrm {B})$ *permits the following decomposition*:

13 $$\begin{aligned} \varvec{\Phi }(\mathrm {B}) = \varvec{\Phi }(1) - (1-\mathrm {B})\dfrac{\varvec{\psi }(\mathrm {B})}{\varvec{\omega }(\mathrm {B})}, \end{aligned}$$

*where* $\varvec{\psi }(\mathrm {B})$ *and* $\varvec{\omega }(\mathrm {B})$ *are polynomial operators, with* $\varvec{\omega }(\mathrm {B})$ *containing no unit root*.

#### Proof

Define the abstract rational function

14 $$\begin{aligned} \varvec{\Omega }(z) = \varvec{\Phi }(\mathrm {1}) - \varvec{\Phi }(z), \end{aligned}$$

where $\varvec{\Phi }(z) = \varvec{\theta }(z) / \varvec{\phi }(z)$ as before. Since $\varvec{\phi }(z)$ has no unit root, $\varvec{\Phi }(1)$ is a finite constant, and it follows that $\varvec{\Omega }(z)$ has no pole at $z=1$. From Eq. (14) it may be seen that $\varvec{\Omega }(1) = 0$. Thus $\varvec{\Omega }(z)$ may be factorized

15 $$\begin{aligned} \varvec{\Omega }(z) = (1-z)\dfrac{\varvec{\psi }(z)}{\varvec{\omega }(z)}, \end{aligned}$$

where $\varvec{\omega }(z)$ contains no unit root. $\square$

#### Theorem

*There exists a* *p*-*vector of coefficients* $(\beta _1, \dots , \beta _p)'$ *such that the linear combination*

16 $$\begin{aligned} r(t) = y(t) - \beta _1 x_1(t) - \dots - \beta _p x_p(t) \end{aligned}$$

*is a stationary time series*. *Specifically*, *r*(*t*) *is stationary when*

17 $$\begin{aligned} \beta _j = \dfrac{1}{\pi _j} \dfrac{\varvec{\Phi }(1)}{\varvec{\Phi }'(1)} \end{aligned}$$

*for all* *j* *in* $1, \dots , p$.

#### Proof

Applying the lemma to Eq. (10) yields

18 $$\begin{aligned} y(t) = \mu + \varvec{\Phi }(1) F(t) - (1-\mathrm {B})\dfrac{\varvec{\psi }(\mathrm {B})}{\varvec{\omega }(\mathrm {B})} F(t) + \varepsilon (t). \end{aligned}$$

Substituting Eq. (8) into (18) gives

19 $$\begin{aligned} y(t)&= \mu + \varvec{\Phi }(1) F(t) - (1-\mathrm {B})\dfrac{\varvec{\psi }(\mathrm {B})}{\varvec{\omega }(\mathrm {B})} \dfrac{1}{1-\mathrm {B}} \Delta F(t) + \varepsilon (t) \end{aligned}$$

20 $$\begin{aligned}&= \mu + \varvec{\Phi }(1) F(t) - \dfrac{\varvec{\psi }(\mathrm {B})}{\varvec{\omega }(\mathrm {B})} \Delta F(t) + \varepsilon (t). \end{aligned}$$

Observe that all terms on the right-hand side are stationary except for $\varvec{\Phi }(1) F(t)$, so the non-stationary component of the forced response is simply a scaled version of the forcing series. This holds similarly for series of model output $x_i$ and their respective forcings. It therefore follows that the non-stationarity in *y* due to forcing $F_i$ may be eliminated by subtracting an appropriately scaled version of the corresponding model output series $x_i$. Expressions for the scaling factors $\beta _i$ in Eq. (17) are readily obtained by considering the relative magnitudes of the non-stationary components of the forced responses. $\square$

Thus it has been established that the optimal fingerprinting regression described in this section is cointegrated, given standard model assumptions, and may be consistently estimated using OLS. The presence of cointegration also renders the OLS estimator superconsistent (Engle and Granger 1987). In practice, the experimental design of a D&A study can be more complicated: GCM simulations are often run with linearly independent combinations of forcing factors, rather than each forcing being applied separately, in order to reduce collinearity of the forced responses (Jones et al. 2016; Jones and Kennedy 2017). Due to the additivity assumption of optimal fingerprinting, the reasoning applied in this section holds similarly in the case of linear combinations of forcings.

### Energy-balance model parameterization

The result presented above holds for a general LTI impulse-response model of the form given in Eq. (4). By choosing a suitable parameterization for the impulse response, this result can be given some physical interpretability. For example, when variable *y* denotes GMST, the impulse response may be parameterized as a *k*-box EBM, which is known to have a discrete-time representation as an ARMA(*k*, $k-1$) filter (Cummins et al. 2020a). In the simplest case, when $k=1$, the EBM reduces to a single ordinary differential equation,

21 $$\begin{aligned} C {\dot{T}}(t) = F(t) -\lambda (T(t) - T_0), \end{aligned}$$

where *T* (K) denotes GMST, $T_0$ (K) is the pre-industrial baseline temperature, *C* (W year m$^{-2}$ K$^{-1}$) is a heat capacity, and $\lambda$ (W m$^{-2}$ K$^{-1}$) is the climate feedback parameter. When $k>1$ the GMST “box” is coupled to a system of additional boxes representing the heat capacity of the deep ocean. Recent studies have identified $k=3$ as the optimal EBM complexity for reproducing the thermal characteristics of recent-generation GCMs (Caldeira and Myhrvold 2013; Tsutsui 2017; Fredriksen and Rypdal 2017; Cummins et al. 2020b). Unfortunately, analytical solutions to *k*-box models quickly become quite complicated, even for $k=2$ (Geoffroy et al. 2013), so in this illustrative example equations are shown for the one-box model only.

If radiative forcing is assumed constant between timesteps, i.e. $F(t) = F(s)$ for $s \in (t-1, t]$, Eq. (21) can be discretized and written in the form of Eq. (10):

22 $$\begin{aligned} T(t) = T_0 + \dfrac{\lambda ^{-1} (1-\mathrm {e}^{-\lambda /C})}{1-\mathrm {e}^{-\lambda /C} \mathrm {B}} F(t), \end{aligned}$$

which may be decomposed into

23 $$\begin{aligned} T(t)&= T_0 + \left[ \dfrac{1}{\lambda } - (1-\mathrm {B}) \dfrac{\lambda ^{-1}{\mathrm {e}^{-\lambda /C}}}{1-\mathrm {e}^{-\lambda /C} \mathrm {B}} \right] F(t) \end{aligned}$$

24 $$\begin{aligned}&= T_0 + \dfrac{F(t)}{\lambda } - \dfrac{\lambda ^{-1}{\mathrm {e}^{-\lambda /C}}}{1-\mathrm {e}^{-\lambda /C} \mathrm {B}} \Delta F(t). \end{aligned}$$

The non-stationary component of *T*(*t*) is simply the input forcing *F*(*t*) scaled by the climate sensitivity $\lambda ^{-1}$, while the stationary component is an AR(1)-filtered version of the forcing increment series $\Delta F(t)$. The AR(1) filter is stationary because $\lambda$ and *C* are both strictly positive. For EBMs with $k>1$ the ARMA filter applied to $\Delta F(t)$ in Eq. (24) will have higher-order polynomials in the numerator and denominator, however the non-stationary component will be unchanged as this term depends only on the climate feedback parameter $\lambda$.

Returning to the question of D&A, let *y*(*t*) and *x*(*t*) denote time series of observed and GCM-simulated historical GMST, driven by forcing series *F*(*t*) and $\pi F(t)$ respectively. If temperature series *y*(*t*) and *x*(*t*) are adequately described by *k*-box EBMs (not necessarily of the same order) with respective climate feedback parameters $\lambda$ and $\lambda '$, then it follows from the theorem that

25 $$\begin{aligned} r(t) = y(t) - \dfrac{\lambda '}{\pi \lambda } x(t) \end{aligned}$$

is a stationary time series. It also follows that the estimator ${\hat{\beta }}_{\mathrm {OLS}}$ obtained by an OLS regression of *y*(*t*) on *x*(*t*) is a superconsistent estimator of $\beta = \lambda '/(\pi \lambda )$ (Engle and Granger 1987).

## Empirical evidence

In the previous section, it was established that, under certain conditions, the variables in optimal fingerprinting regression are provably cointegrated, implying consistency of least-squares parameter estimation. Though cointegration is predicted by the theory, whether it arises in reality will depend on the validity of the model assumptions. Two of the main assumptions used to obtain results in Sect. 3 are standard in optimal fingerprinting:

1. additivity, that the combined effect of multiple forcing factors is the sum of their effects had they been applied separately;
2. correct forcing specification, that radiative forcings in GCMs have correct temporal structure up to a multiplicative constant.

Identifying these assumptions as necessary prerequisites for cointegration of GCM output and historical observations allows them to be assessed using numerical cointegration tests. If there is strong numerical evidence of cointegration, then this gives no reason to doubt the validity of assumptions 1 and 2, and by extension the consistency of optimal fingerprinting. On the other hand, should significant cointegration fail to be detected, then the possibility of violated assumptions, spurious regression and meaningless results cannot be discounted without further investigation.

Two further assumptions were made in Sect. 3 which are non-standard in optimal fingerprinting. Firstly, it was assumed that non-stationary forcings are I(1), for reasons set out in Sect. 1. Since the true underlying forcing series are not directly observable, it is infeasible to assess this assumption directly. However, as with the standard optimal fingerprinting assumptions above, the idealized concept of I(1) forcings may be shown “not inconsistent” with observation in the event that significant cointegration is detected. The second non-standard assumption is that of LTI impulse responses, which may be seen as a strengthening of the standard additivity assumption. To limit the influence of this strengthening, numerical results in this section have been calculated using the GMST climate variable, whose response to a radiative forcing perturbation is known to be well-modelled using LTI impulse responses (Li and Jarvis 2009; Good et al. 2011; Geoffroy et al. 2013).

### Data

The numerical analyses in this section were performed using observed and GCM-simulated time series of GMST, averaged annually (Jan–Dec) for the period 1880–2014.

Observational datasets were, in alphabetical order: Berkeley Earth (Rohde and Hausfather 2020), Cowtan and Way 2.0 (Cowtan and Way 2014), GISTEMP v4 (Lenssen et al. 2019; GISTEMP Team 2021), HadCRUT5 (Morice et al. 2021) and NOAAGlobalTemp V5 (Smith et al. 2008; Huai-Min Zhang et al. 2019). The choice of observational dataset was found not to affect hypothesis test results. The results presented here were calculated using HadCRUT5, however the whole analysis may be re-run for the other observational datasets by changing a single line of code (see Data Availability statement for details).

Predicted climate change signals were calculated using simulation output from 13 GCMs of the CMIP6 generation (Eyring et al. 2016). Chosen models are from modelling centres who have contributed runs as part of the DAMIP project (Gillett et al. 2016). For each GCM, ensemble-mean annual-GMST time series were calculated for the *historical* and *hist-GHG* experiments. These two forcing scenarios were chosen because GHG-attributable warming is of primary interest. Jones et al. (2016) recommend a two-way attribution of this form on the grounds of robustness. Table 1 gives the respective sizes of the *historical* and *hist-GHG* ensembles for each GCM, as well as the corresponding model citations.

**Table 1:** CMIP6 GCM ensemble sizes and citations

Model	# *historical*	# *hist-GHG*	Citation
ACCESS-ESM1-5	20	3	Ziehn et al. (2020)
BCC-CSM2-MR	3	3	Wu et al. (2019)
CanESM5	65	50	Swart et al. (2019)
CESM2	11	3	Danabasoglu et al. (2020)
CNRM-CM6-1	30	10	Voldoire et al. (2019)
FGOALS-g3	6	3	Li et al. (2020)
GFDL-ESM4	3	1	Dunne et al. (2020)
GISS-E2-1-G	46	10	Kelley et al. (2020)
HadGEM3-GC31-LL	4	4	Williams et al. (2018)
IPSL-CM6A-LR	32	10	Boucher et al. (2020)
MIROC6	50	3	Tatebe et al. (2019)
MRI-ESM2-0	7	5	Yukimoto et al. (2019)
NorESM2-LM	3	3	Seland et al. (2020)

### Cointegration tests

Let *y* denote observed historical GMST and let $x_1,x_2$ denote GCM-predicted signals corresponding to the *historical* and *hist-GHG* experiments respectively. The theory in Sect. 3 predicts that the time series $y(t), x_1(t), x_2(t)$ are cointegrated. A simple test for cointegration consists of fitting the linear regression model

26 $$\begin{aligned} y(t) = \beta _0 + \beta _1 x_1(t) + \beta _2 x_2(t) + \varepsilon (t) \end{aligned}$$

using OLS and then testing the series of residuals ${\hat{\varepsilon }}(t)$ for stationarity (Engle and Granger 1987). Residual stationarity may be tested using the procedure of Dickey and Fuller (1979), whereby the autoregression

27 $$\begin{aligned} \Delta {\hat{\varepsilon }}(t) = \delta _0 + \delta _1 {\hat{\varepsilon }}(t-1) + u(t) \end{aligned}$$

is estimated using OLS and the *t*-statistic corresponding to ${\hat{\delta }}_1$, denoted ${\hat{\tau }}_c$, compared with a relevant quantile of the reference “Dickey–Fuller” distribution. The null hypothesis $H_0$ is that no cointegrating relationship exists between $y(t), x_1(t), x_2(t)$ and the residuals exhibit unit-root non-stationarity. A significant negative estimate ${\hat{\delta }}_1$ provides evidence of mean-reverting residuals and leads to a rejection of $H_0$ in favour of the alternative hypothesis: that the residuals are stationary and series $y(t), x_1(t), x_2(t)$ cointegrate. The appropriate reference distribution depends on the number $N=3$ and length $n=135$ of potentially I(1) time series being regressed in Eq. (26). Using the third-order approximation formula in MacKinnon (2010), the critical value for a cointegration test at the one-percent level is $\tau _c = -4.40$. It should be noted that the I(1) assumption refers to the degree of differencing required to achieve stationarity and is best regarded as an upper bound on the level of trendiness anywhere in the time series. The above cointegration test is therefore also valid for I(0) data: the results would just be more conservative.

**Fig. 1:**
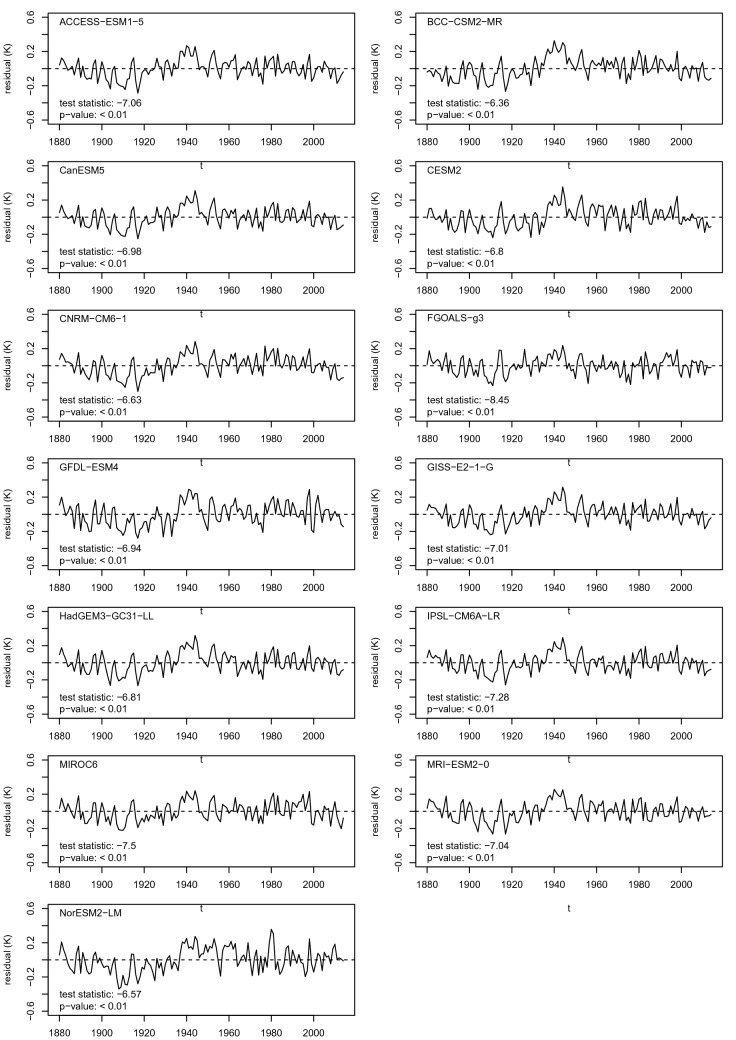
Cointegration test results. Time series are residuals from two-way OLS regressions of HadCRUT5 GMST observations on GCM output from *historical* and *hist-GHG* experiments. Test statistics $<-4.40$ are significant at the one-percent level, indicating residual stationarity and cointegration

Tests of this form were performed for combinations of the HadCRUT5 historical observations with output from each of the 13 CMIP6 GCMs considered in this study. Figure 1 shows time series of residuals ${\hat{\varepsilon }}(t)$ from the fitted regression in Eq. (26), with test statistics ${\hat{\tau }}_c$ and associated p-values. It can be seen from the residual plots that all 13 time series exhibit strong mean-reverting behaviour. This is confirmed by the results of the cointegration tests: the null hypothesis of “no cointegration” was rejected at the one-percent level for all 13 GCMs.

The residual time series in Fig. 1 share common features, such as an apparent bump around the year 1940. This is as expected. From the theorem in Sect. 3 it follows that the regression residuals in Eq. (26) include contributions from the stationary components of the forced trends in $y(t), x_1(t), x_2(t)$ (i.e. those terms involving $\Delta F(t)$), as well as from internal climate variability in those series. Since the GCMs have similar impulse responses, and since the realization of internal variability in HadCRUT5 is common to all 13 regressions, the only truly independent contribution to each residual series comes from that GCM’s realizations of internal variability.

### Attribution of surface temperature warming

Having detected signficant cointegration of $y(t), x_1(t), x_2(t)$, it follows that the coefficients $\varvec{\beta } = (\beta _1, \beta _2)'$ in Eq. (26) may be consistently estimated using OLS. However, the regression residuals (see Fig. 1) are serially correlated, meaning that the usual formulas for calculating standard errors and confidence regions are invalid. Optimal fingerprinting studies commonly address this problem by estimating the covariance structure of internal climate variability from a GCM’s piControl simulation (Allen and Tett 1999). This approach ignores the stationary forced component of the residuals, which arises due to differences between the impulse responses of GCMs and the true climate. Using piControl also relies on GCMs accurately simulating the pre-industrial climate, which cannot be verified through observation. The use of piControl for hypothesis testing in optimal fingerprinting has recently been criticized (McKitrick 2021).

An alternative way to avoid the problem of serially correlated residuals, without introducing dependence on piControl simulations, is to fit a dynamic regression model which includes lagged versions of time series $y(t), x_1(t), x_2(t)$ (Hendry and Juselius 2000). Fitting dynamic regressions of the form

28 $$\begin{aligned} y(t)= & {} \beta _0' + \beta _1' x_1(t) + \beta _2' x_2(t) + \beta _3' y(t-1) + \beta _4' x_1(t-1) \nonumber \\&+ \beta _5' x_2(t-1) + \varepsilon '(t) \end{aligned}$$

using OLS yields serially uncorrelated residual series for each of the 13 GCMs. The usual normal distribution theory may then be assumed to hold asymptotically for estimates of the coefficients $\varvec{\beta }'=(\beta _1',\dots ,\beta _5')'$ in Eq. (28).

Dynamic regression coefficients $\varvec{\beta }'$ in Eq. (28) can be related back to coefficients $\varvec{\beta }$ in Eq. (26) via the Granger representation theorem, which requires that systems of cointegrated series have equivalent representations as error-correction models (ECMs) (Engle and Granger 1987). Observe that Eq. (28) may be written

29 $$\begin{aligned} \Delta y(t)= & {} \beta _1' \Delta x_1(t) + \beta _2' \Delta x_2(t) \nonumber \\&- \alpha \left[ y(t-1) - \beta _0 - \beta _1 x_1(t-1) - \beta _2 x_2(t-1) \right] \nonumber \\&+ \varepsilon '(t), \end{aligned}$$

where $\alpha =1-\beta _3'$, $\beta _0=\beta _0'/\alpha$, $\beta _1=(\beta _1'+\beta _4')/\alpha$, and $\beta _2=(\beta _2'+\beta _5')/\alpha$. The expression inside the square brackets, called the error-correction term, is a stationary linear combination of $y(t), x_1(t), x_2(t)$. By estimating the dynamic regression model in Eq. (28) using OLS, and then reparameterizing to obtain the ECM in Eq. (29), it is possible to recover estimates of coefficients $\varvec{\beta }$ in Eq. (26).

Because the *historical* CMIP6 experiment includes GHG forcing, parameters $\beta _1$ and $\beta _2$ must be transformed to obtain the scaling factors of primary interest. Following the notation of Jones et al. (2016), let $\beta _{\mathrm {G}}=\beta _1 + \beta _2$ and $\beta _{\mathrm {OAN}}=\beta _1$ denote the scaling factors to be applied to GCM-predicted signals forced by GHG emissions and “other anthropogenic and natural” factors respectively. Although $\varvec{\beta }^* = (\beta _{\mathrm {G}},\beta _{\mathrm {OAN}})'$ is linearly related to coefficients $\varvec{\beta }$, the function relating $\varvec{\beta }$ to $\varvec{\beta }'$ is non-linear. The partially linear function $f: \varvec{\beta }' \mapsto \varvec{\beta }^*$ may be written

30 $$\begin{aligned} f(\varvec{\beta }') = g(\varvec{\beta }') h(\varvec{\beta }'), \end{aligned}$$

where $g(\varvec{\beta }') = (1-\beta _3')^{-1}$ is the non-linear part of *f*, and $h(\varvec{\beta }') = M \varvec{\beta }'$ is the linear part where

31 $$\begin{aligned} M = \begin{pmatrix} 1 &{} 1 &{} 0 &{} 1 &{} 1 \\ 1 &{} 0 &{} 0 &{} 1 &{} 0 \end{pmatrix}. \end{aligned}$$

Let

32 $$\begin{aligned} J(\varvec{\beta }') = h(\varvec{\beta }') \nabla g(\varvec{\beta }')' + \dfrac{\partial h(\varvec{\beta }')}{\partial \varvec{\beta }'} g(\varvec{\beta }') \end{aligned}$$

denote the Jacobian of *f*. If coefficient estimates $\hat{\varvec{\beta }}'$, obtained by fitting Eq. (28) using OLS, have estimated covariance ${\hat{\Sigma }}'$, then a linearized estimate of the covariance of $\hat{\varvec{\beta }}^* = f(\hat{\varvec{\beta }}')$ is given by ${\hat{\Sigma }}^* = J(\hat{\varvec{\beta }}') {\hat{\Sigma }}' J(\hat{\varvec{\beta }}')'$. An approximate 90% confidence ellipse for $\varvec{\beta }^*$ satisfies

33 $$\begin{aligned} (\hat{\varvec{\beta }}^* - \varvec{\beta }^*)' \left( {\hat{\Sigma }}^* \right) ^{-1} (\hat{\varvec{\beta }}^* - \varvec{\beta }^*) < 2 F_{2,128} (0.90), \end{aligned}$$

where $F_{2,128} (0.90)$ denotes the 90th percentile of the *F* distribution with degrees of freedom two and 128. Residual degrees of freedom are 135 (years of observations from 1880 to 2014), minus one (due to differencing), minus six (parameters estimated to fit Eq. (28)), giving 128.

**Fig. 2:**
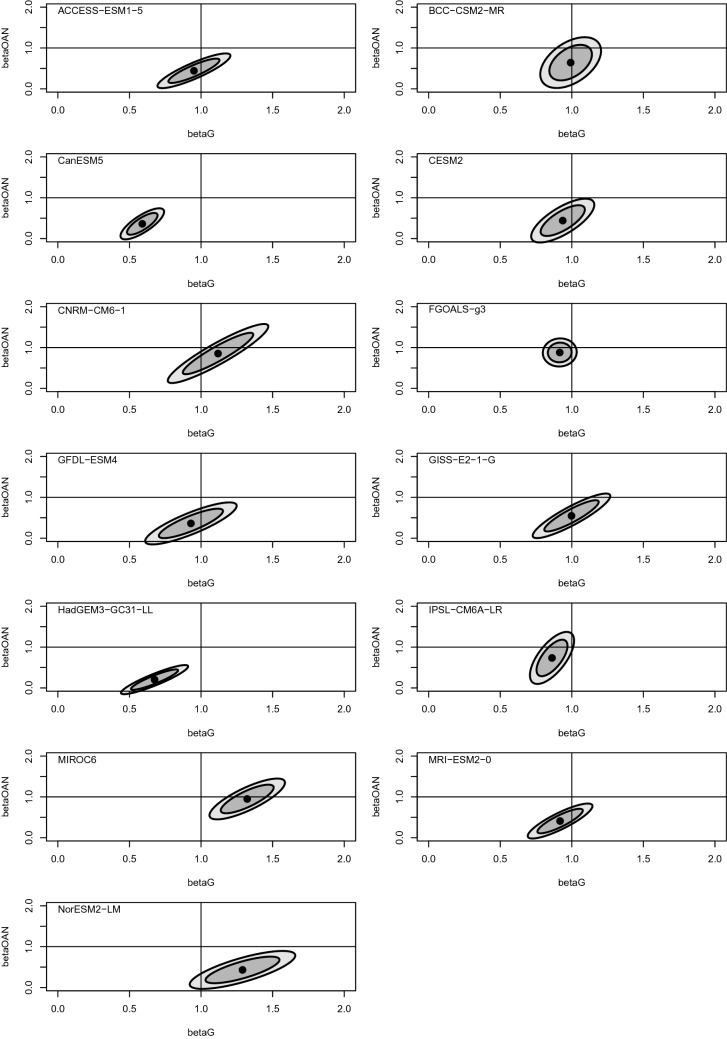
Scaling factor confidence ellipses. Black dots are point estimates of scaling factors ${\hat{\beta }}_{\mathrm {G}},{\hat{\beta }}_{\mathrm {OAN}}$ for each GCM, obtained using dynamic OLS regression. Smaller and larger shaded ellipses are approximate 90% and 99% confidence regions respectively

**Table 2:** Estimated scaling factors and attributable warming

Model	${\hat{\beta }}_{\mathrm {G}}$	(s.e.)	${\hat{\beta }}_{\mathrm {OAN}}$	(s.e.)	$\Delta T_{\mathrm {total}}$ (K)	$\Delta T_{\mathrm {GHG}}$ (K)
ACCESS-ESM1-5	0.95	(0.083)	0.44	(0.14)	1.02	1.22
BCC-CSM2-MR	0.99	(0.069)	0.64	(0.20)	0.99	1.04
CanESM5	0.59	(0.049)	0.36	(0.12)	1.01	1.18
CESM2	0.94	(0.071)	0.44	(0.17)	1.05	1.14
CNRM-CM6-1	1.12	(0.114)	0.85	(0.23)	1.08	1.47
FGOALS-g3	0.92	(0.038)	0.88	(0.11)	1.02	0.93
GFDL-ESM4	0.93	(0.104)	0.36	(0.17)	1.05	1.20
GISS-E2-1-G	1.00	(0.088)	0.55	(0.18)	1.04	1.28
HadGEM3-GC31-LL	0.68	(0.076)	0.20	(0.12)	1.05	1.22
IPSL-CM6A-LR	0.86	(0.050)	0.73	(0.21)	1.07	1.18
MIROC6	1.32	(0.085)	0.95	(0.16)	1.05	1.29
MRI-ESM2-0	0.92	(0.073)	0.41	(0.14)	1.04	1.25
NorESM2-LM	1.29	(0.119)	0.43	(0.15)	1.02	1.20

Point estimates of ${\hat{\beta }}_{\mathrm {G}}$ and ${\hat{\beta }}_{\mathrm {OAN}}$ are obtained from OLS fits of the dynamic regression model in Eq. (28) to HadCRUT5 GMST observations. Reported standard errors are calculated by linearizing $f: \varvec{\beta }' \mapsto \varvec{\beta }^*$ about $\varvec{\beta }' = \hat{\varvec{\beta }}'$. Columns $\Delta T_{\mathrm {total}}$ and $\Delta T_{\mathrm {GHG}}$ are corresponding estimates of the total and GHG-attributable increases in GMST between the reference periods 1880–1899 and 2005–2014, obtained by appropriate scaling of GCM-predicted signals

**Fig. 3:**
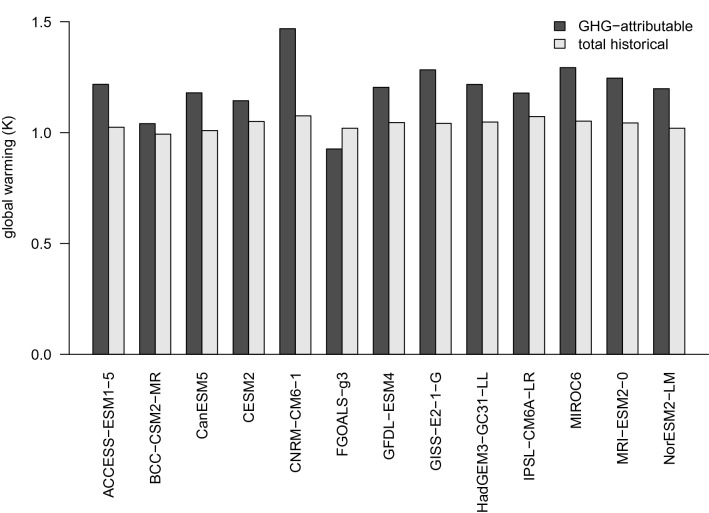
Estimated total and GHG-attributable increases in GMST between the reference periods 1880–1899 and 2005–2014, obtained by appropriate scaling of GCM-predicted signals

Point estimates and confidence ellipses have been calculated for scaling factors $\beta _{\mathrm {G}}$ and $\beta _{\mathrm {OAN}}$ using the methodology described above (see Fig. 2 and Table 2). Collinearity between time series $y(t), x_1(t), x_2(t)$ and their lagged counterparts means that, taken individually, coefficient estimates $\hat{\varvec{\beta }}'$ are subject to greater uncertainty than the classical OLS estimates $\hat{\varvec{\beta }}$. However, the estimates ${\hat{\beta }}_{\mathrm {G}}$ and ${\hat{\beta }}_{\mathrm {OAN}}$ obtained by back-transforming $\hat{\varvec{\beta }}'$ are well-constrained and closely resemble the classical estimates (proportional change has mean 0.008 and standard deviation 0.07). The inflation of parameter uncertainty which results from including lagged variables is a necessary consequence of accounting for residual autocorrelation. Scaling factor standard errors vary across different GCMs. This is partly a consequence of variation in the size of available ensembles, with increased ensemble size leading to smaller standard errors. Standard errors are also affected by the GCMs’ ability to reproduce the forced patterns in the observations. Table 2 also includes estimates of attributable warming between the reference periods 1880–1899 and 2005–2014. Attributable warming was calculated by first computing the mean difference in GMST between the reference periods for each of the *historical* and *hist-GHG* GCM experiments, and then taking appropriate linear combinations of the temperature differences with coefficients determined by the relevant scaling factors. These results are visualized in Fig. 3, where total historical and GHG-attributable warming are plotted side-by-side. From Fig. 3 it may be seen that for 12 out of 13 CMIP6 GCMs GHG-attributable warming exceeds total historical warming.

There exist some differences between attribution results in the present study and those of Gillett et al. (2021) and Chapter 3 of IPCC AR6 (Eyring et al. 2021). For example, in Table 1 of Gillett et al. (2021), the authors of that paper report GHG-attributable warming of 1.16–1.95 degrees in 2010–2019 relative to an 1850–1900 reference period, whereas the point estimates of GHG-attributable warming obtained in the present study range from 0.93 to 1.47. The likely range given in AR6 is 1.0–2.0 degrees. While the attribution of surface warming performed in this section demonstrates how cointegration theory may be applied to uncertainty quantification for scaling factors in D&A of climate trends, rigorous assessment of the new method’s performance and true “apples-to-apples” comparison with current IPCC estimates will require a full suite of “perfect-model” numerical experiments. Such numerical experiments have recently been employed for testing coverage rates of confidence intervals in optimal fingerprinting (Li et al. 2021).

## Summary

Optimal fingerprinting, the statistical methodology commonly used for D&A of climate change trends, is typically performed by linearly regressing non-stationary climate variables. Non-stationary time series regressions are, in general, statistically inconsistent and liable to produce spurious results. This study has shown, by modelling radiative forcing as an integrated stochastic process within an idealized linear-response-model framework, that the optimal fingerprinting estimator is consistent under standard D&A assumptions. Hypothesis tests, combining observations of historical GMST with simulation output from 13 CMIP6-generation GCMs, produce no evidence that standard assumptions have been violated. It is therefore concluded that, at least in the case of GMST, detection and attribution of climate change trends is very likely not spurious regression. Furthermore, detection of significant cointegration between observations and GCM output indicates that the OLS estimator is superconsistent, with better convergence properties than might previously have been assumed. Finally, a new method has been developed for quantifying D&A uncertainty, which exploits the notion of cointegration to eliminate the need to rely on piControl GCM simulations and the corresponding strong assumptions.

## Data Availability

The datasets and code used to perform the analyses in this study are available online at https://doi.org/10.5281/zenodo.5008827. Code is written in the statistical programming language R (R Core Team 2021). The ellipses in Fig. 2 were plotted using the “ellipse” function from the *car* package by Fox et al. (2011).
